# Connective Tissue Growth Factor From Periosteal Tartrate Acid Phosphatase-Positive Monocytes Direct Skeletal Stem Cell Renewal and Fate During Bone Healing

**DOI:** 10.3389/fcell.2021.730095

**Published:** 2021-09-14

**Authors:** Yun Bai, Tao Yu, Jiezhong Deng, Yusheng Yang, Jiulin Tan, Qijie Dai, Zehua Zhang, Shiwu Dong, Jianzhong Xu

**Affiliations:** ^1^Department of Orthopedics, Southwest Hospital, Third Military Medical University, Chongqing, China; ^2^Department of Biomedical Materials Science, School of Biomedical Engineering, Third Military Medical University, Chongqing, China

**Keywords:** skeletal stem cell, ctgf, TRAP-positive monocytes, fracture healing, bone regeneration

## Abstract

The periosteum is critical for bone healing. Studies have shown that the periosteum contains periosteal stem cells (PSCs) with multidirectional differentiation potential and self-renewal ability. PSCs are activated in early fracture healing and are committed to the chondrocyte lineage, which is the basis of callus formation. However, the mechanism by which PSCs are activated and committed to chondrocytes in bone regeneration remains unclear. Here, we show that tartrate acid phosphatase (TRAP)-positive monocytes secrete CTGF to activate PSCs during bone regeneration. The loss function of TRAP-positive monocytes identifies their specific role during bone healing. Then, the secreted CTGF promotes endochondral ossification and activates PSCs in mouse bone fracture models. The secreted CTGF enhances PSC renewal by upregulating the expression of multiple pluripotent genes. CTGF upregulates c-Jun expression through αVβ5 integrin. Then, c-Jun transcription activates the transcription of the pluripotent genes *Sox2*, *Oct4*, and *Nanog*. Simultaneously, CTGF also activates the transcription and phosphorylation of Smad3 through αVβ5 integrin, which is the central gene in chondrogenesis. Our study indicates that TRAP-positive monocyte-derived CTGF promotes bone healing by activating PSCs and directing lineage commitment and that targeting PSCs may be an effective strategy for preventing bone non-union.

## Introduction

Bones are repeatedly and routinely healed wounds or acute injuries for regeneration, which is different from many other tissues that repair scars (Vi et al., 2018). However, the healing process is not completely flawless and sometimes fails, resulting in a delayed healing rate of 5–10% (Hu et al., 2017). In clinical practice, approximately one-sixth patients exhibit fracture non-union, which results in a huge physical and economic burden on the affected individuals and the society. Fracture non-union or delayed union may occur due to many reasons, including infection at the fracture site, insufficient blood supply to the bone, and insufficient bone stability. These factors hinder effective bone callus formation owing to the limited ability of cartilage generation. Therefore, studying the mechanism of fracture healing is crucial for solving the problem of failure of fracture healing.

Endochondral ossification is the primary means of bone regeneration in bone defects (Raggatt et al., 2014). Bone marrow mesenchymal stem cells (BMSCs) were believed to be the cellular source of endochondral ossification. Although BMSCs are widely used in bone tissue engineering, evidence suggests that BMSCs are not the main cellular origin of bone callus during fracture healing. On the other hand, the periosteum directly participates in the maintenance of bone integrity. Furthermore, the periosteum is crucial for typical fracture healing (Ho-Shui-Ling et al., 2018; Li et al., 2019). Studies have shown that the periosteum contains periosteal stem cells (PSCs) that possess higher ability to repair bones. The PSC is a population of high-purity skeletal stem cells (SSCs) with multipotent capacity, which can differentiate into bone, cartilage, and bone marrow cells (Chan et al., 2015, 2018). Compared with BMSCs, PSCs, which are activated in the early stage of bone regeneration, have enhanced cell growth and clonal formation capabilities and excellent regeneration capabilities, which are fundamental to bone regeneration. For lineage tracing, MSCs were labeled with GFP and transferred into mice with bone injury (Duchamp De Lageneste et al., 2018). The transplanted PSCs merged into the callus at its center, but a majority of the BMSCs remained at the periphery of the callus. These results indicate that PSCs contribute more to callus formation than BMSCs. Another study revealed that no PSC-derived chondrocytes were observed at the baseline. However, when a fracture occurs, PSCs contribute to approximately half of the cartilage tissue in the fractured callus. In a renal capsule transplantation model, PSCs isolated from the bone callus differentiated into cartilage tissue (Debnath et al., 2018), indicating that the fate of PSCs in bone repair shifts from osteoblast to chondrocyte differentiation and that PSCs explain this functional transformation of the periosteum by converting the ability of intramembranous bone formation at baseline into the ability to obtain endochondral bone formation after a fracture. However, how PSCs are activated and committed to the chondrocyte lineage during bone regeneration remains unclear.

Fracture healing occurs in a microenvironment where various cells cooperate and interact with each other in different ways. In the process of bone remodeling, osteoclast precursors migrate and differentiate because of endocrine and paracrine factors. This process is accompanied by both bone resorption by osteoclasts and bone formation by osteoblasts (Tang et al., 2009). After responding to the stimulation of M-CSF and RANKL, monocytes and macrophages first commit to the osteoclast lineage (mononuclear preosteoclast), which is characterized by resistance to tartrate acid phosphatase (tartrate-resistant acid phosphatase; TRAP^+^). Subsequently, fusion of the preosteoclasts leads to the formation of TRAP-positive mature osteoclasts. These osteoclasts are multinucleated and possess bone resorption capacity. Studies have shown that TRAP-positive monocytes reside in the periosteum and secrete PDGF-BB during bone modeling. This PDGF-BB induces angiogenesis coupled with osteogenesis by S1P signaling (Xie et al., 2014). TRAP-positive monocyte-conditioned medium induces the migration of MSCs and endothelial progenitor cells (EPCs) and enhances EPC tube formation. Results of the aforementioned studies and the existing regulation of bone remodeling by TRAP-positive monocytes suggest that TRAP-positive monocytes may participate in PSC activation.

CTGF, a member of the CCN protein family, can promote proliferation, adhesion, migration of multiple types of cells, as well as angiogenesis (Carlstrom et al., 2015). CTGF participates in endochondral ossification by acting on many types of cells (Takigawa, 2013). CTGF expression is also important for chondrocyte hypertrophy to form a calcified matrix. CTGF overexpression promoted growth plate growth and increased the length of long bones in transgenic mice (Arnott et al., 2011). Research has also proven that CTGF is the main regulator of cartilage formation. The multidomain feature allows CTGF to interact with other growth factors such as TGF-β, BMP, IGF, and VEGF (Shen et al., 2021). Simultaneously, CTGF interacts with the extracellular matrix and cell surface receptors, such as integrins (Perbal, 2004).

This study reported that TRAP-positive monocytes secrete CTGF to activate PSCs and direct chondrocyte lineage commitment during fracture healing. First, the number and distribution of TRAP-positive monocytes were identified in the periosteum after injury. We then found that TRAP-positive monocytes secrete CTGF to promote endochondral ossification and PSC renewal in bone callus. The mechanism by which CTGF derived from TRAP-positive monocytes acts on PSCs was also elucidated.

## Materials and Methods

### Mice

Rosa26-iDTR (*DTR*^*fl/fl*^) mice (#007900) and tdTomato reporter mice (#007909) were purchased from Jackson laboratory (United States). Acp5-Cre mice were purchased from GemPharmatech Co., Ltd (Chengdu, China). *Ccn2^*fl/fl*^* mice–bearing loxP sites flanking exon 4 of the cellular communication network factor 2 gene were also purchased from Jackson laboratory (# 035182). All mice were bred under SPF conditions in the Laboratory Animals Center of Army Medical University (Third Military Medical University, Chongqing, China). Age- and sex-matched littermates were used as control mice. All experiments were conducted according to the Third Military Medical University Sciences Guide for Laboratory Animals.

### Primary Cultures of Periosteal Stem Cells

The mice were sacrificed and their femurs and tibias were dissected. After removing the epiphysis, the bone was flushed for removing total bone marrow cells. To obtain primary PSCs, the remaining bone-washed explants without muscles and tendons were cultured using Mouse MesenCult^TM^ Expansion Kit (# 05513, STEMCELL Technologies, United States) supplemented with L-Glutamine (# 07100, STEMCELL Technologies); the PSCs migrated from the explants within 3 days. After 2 weeks, the bones were removed, and the PSCs were digested with trypsin and directly used for *in vitro* and *in vivo* experiments without further amplification.

### Colony-Forming Efficiency Assay and Differentiation of Periosteal Stem Cells *in vitro*

PSCs obtained were directly plated at a density of 1,000 cells/cm^2^ in stem cell growth media (# 05513, STEMCELL Technologies) for 2 weeks, with the medium being changed every 3 days. Clones were fixed in 4% paraformaldehyde for 0.5 h, stained with Giemsa stain (# G5637, Sigma-Aldrich, United States), and counted.

For chondrogenic differentiation, cells were grown in micromass culture. About 5 × 10^5^ PSCs were plated in the center of a culture plate and incubated at 37°C with 5% CO_2_ for 3 h. Without disturbing the pellet, the chondrogenic medium (# MUBMX-9004, Cyagen, China) was carefully added to the cells. The cell micromass was fixed in 4% paraformaldehyde for 0.5 h on day 14. Alcian blue staining (Cyagen) was performed to demonstrate the presence of matrix proteoglycans. The stain was then extracted for quantification through measurement of absorbance at 620 nm.

For osteogenic differentiation, the PSCs were treated with an osteogenic differentiation medium kit (# MUBMD-90021, Cyagen) in accordance with the manufacturer’s instructions. Mineralized nodules were stained with alizarin red solution in osteogenic differentiation medium kit. This staining procedure was quantitatively analyzed by measuring absorbance at 405 nm.

In some experiments, the Src inhibitor PP2 (10 μM, # P0042, Sigma–Aldrich), anti-integrin αvβ5 (1 μg/mL, # MAB1961, Sigma–Aldrich), and recombinant CTGF (rCTGF, 100 ng/mL, # sc-515116, Santa Cruz) were added to the medium.

### Preparation of Osteoclast Conditioned Medium

The bone marrow of the femur and tibia of 4-week-old wild-type male mice were washed to collect monocytes and macrophages (BMMs). The washed bone marrow cells were cultured in α-MEM culture media containing 10% FBS, 100 U/mL penicillin, 100 μg/mL streptomycin sulfate, and 30 ng/mL M-CSF (# 416-ML-010/CF, R&D, United States) for 6 h. After the adherent cells were discarded, the floating cells were incubated with α-MEM culture media containing M-CSF (30 ng/mL) to obtain pure BMMs. When the BMMs were incubated in a 12-well plate (2 × 10^5^ cells/well) containing 30 ng/mL M-CSF and 50 ng/mL RANKL (# 462-TEC-010, R&D), all cells became precursor osteoclasts after 3 days of culture. The incubation was continued with 30 ng/mL M-CSF and 100 ng/mL RANKL for 7 days to form mature multinucleated osteoclasts. Then, the conditioned media was harvested from preosteoclasts and mature osteoclasts. After centrifugation (2,500 r.p.m., 10 min at 4°C), the conditioned medium was aliquoted and stored at −80°C. In subsequent experiments, neutralizing antibodies for certain factors, including IGFBP5 (# sc-515116, Santa Cruz, 1 μg/mL), CXCL12 (# sc-74271, Santa Cruz, 1 μg/mL), and CTGF (# sc-365970, Santa Cruz, 1 μg/mL), were added to the conditioned media for 3d (CFE) or for 7d (chondrogenesis).

### Flow Cytometry

We harvested the fractured callus of wild-type or Ccn2Acp5 mice on the 14th day after the fracture. Using a mortar and pestle, each sample was crushed separately and placed in separate 50-ml centrifuge tubes with 10 mL collagenase at 37°C for 30 min Each sample was subjected to continuous enzymatic digestion with collagenase and DNase at 37°C with gentle shaking for 30 min. The digestion step was repeated thrice. The digested cells were filtered through a 70-μm nylon mesh, centrifuged at 200 × g at 4°C, and resuspended in PBS containing 2% FBS. After the cells were washed with PBS, they were resuspended in 50 μL PBS containing 2% FBS. The pellet was stained using fluorescent dye-conjugated antibodies against CD45 (1:100, # 147718, Biolegend, United States), Tie2 (1:100, # 124009, Biolegend), CD51 (alpha V, 1:100, # 104105, Biolegend), Ly-51 (6C3, 1:100, # 108314, Biolegend), Ter119 (1:100, # 116228, Biolegend), CD105 (1:100, # 120406, Biolegend), CD200 (1:50, # 565547, BD Biosciences, United States), and Thy (CD90.2, 1:100, # 105335, Biolegend) for flow cytometry analysis.

### Plasmids and siRNA

To construct the dual-luciferase reporter vector, the 1-kb upstream promoter regions of Jun and Smad3 were cloned into the pLG3 vector (Promega, Madison, WI, United States). Luciferase activity was detected using the double Luciferase Assay System (Promega) and normalized with the co-expressed Renilla luciferase. siRNAs of c-Jun (# sc-29224, Santa Cruz, United States) and Smad3 (# sc-37239, Santa Cruz) were purchased from Santa Cruz Biotechnology.

### Mice Bone Fracture Model and Periosteum Grafting

Eight-week-old male or female mice were used for the model. The mice were anaesthetized with 0.5% sodium pentobarbital (# P3761, Sigma-Aldrich). A closed, transverse fracture was made in the tibia by three-point bending, which was confirmed by radiography. Periosteum grafts isolated from the tibia tdTomato donor mice were transplanted. Briefly, we anaesthetized wild-type or *Ccn2^*Acp*5^* mice to make open and unstable fractures. Then, we carefully scraped off the two tibial periosteums of a tdTomato donor using microdissection surgical instruments and collected and transplanted them at the site of non-stabilized tibial fractures in 8-week-old *Ccn2^*fl/fl*^* or *Ccn2^*Acp*5^* host mice. The muscle was then sutured to fix the grafted periosteum and then the skin was closed.

### Micro-Computed Tomography Analysis

Tibia specimens from different mice groups were fixed overnight in 4% paraformaldehyde. The specimens were scanned using micro-computed tomography (μCT; Skyscan1272, Bruker microCT, Kontich, Belgium). The scanner was set to a voltage of 60 kV and a resolution of 8 μm per pixel. NRecon v1.6 software (Bioz, Inc., United States) was used to reconstruct the scanned image. The reconstruction was analyzed using CTAn v1.9 software (Bruker micro-CT), and CTVol v2.0 software (Bruker micro-CT) was used to visualize the 3D model. The area of interest was defined according to the “fracture callus analysis” section of Bruker micro-CT method annotation.

### Histochemistry

The sample was analyzed by CT and then decalcified using 0.5 M EDTA decalcification solution for a week at 25°C. The samples are embedded in paraffin. Using a paraffin microtome, 4-μm-thick bone sections were prepared for TRAP staining (# 387A-1KT, Sigma-Aldrich) and safranin O (# S2255, Sigma-Aldrich)/fast green (# F7252, Sigma-Aldrich) staining. For safranin O/fast green staining, the sections were dewaxed and washed thrice with phosphate-buffered saline (PBS). The sections were stained with fast green for 5 min, followed by differentiation with 1% acetic acid for 10 s. Subsequently, the sections were counterstained with safranin O for 5 min. For TRAP staining, the TRAP staining solution was first prepared according to the manufacturer’s instructions. The sections were dewaxed, washed thrice with PBS, stained with TRAP staining solution for 5 min at 70°C, and counterstained with methyl green (#M884, Sigma-Aldrich) for 10 s.

### Real-Time Quantitative PCR

For total RNA extraction, RNAiso Plus reagent (# 91089, Takara, Japan) was used. cDNA was prepared from 1 μg of total RNA using PrimeScript^TM^ RT reagent Kit with gDNA Eraser (# RR047B, Takara), according to the manufacturer’s instructions. PCR amplifications were performed using specific primers for each gene as follows: *Sox2* (F) 5′-G CGGAGTGGAAACTTTTGTCC- 3′, (R) 5′-CGGGAAGCGT GTACTTATCCTT-3′; *Oct4* (F) 5′-GGCTTCAGACTTCGCCT CC-3′, (R) 5′-AACCTGAGGTCCACAGTATGC-3′; *Nanog* (F) 5′ -TCTTCCTGGTCCCCACAGTTT-3′, (R) 5′-GCAAGAATAG TTCTCGGGATGAA-3′; *Sox9* (F) 5′-GAGCCGGATCTGAAG AGGGA-3′, (R) 5′-GCTTGACGTGTGGCTTGTTC-3′; *Col2a1* (F) 5′-GGGAATGTCCTCTGCGATGAC-3′, (R) 5′-CAGGC GCACCATCTCTGAT-3′.

### Western Blots and Antibodies

Cells were lysed in cell lysis buffer (#P0013, Beyotime Biotechnology, China). In total, 20 μg of protein samples was subjected to SDS-PAGE. Next, the proteins were transferred onto PVDF membranes (#ISEQ00010, Merck Millipore, Germany). Then, the membranes were blocked in 5% skim milk for 2 h and incubated with primary antibodies overnight at 4°C. The primary antibodies for GAPDH (1:1,000, # 5,174), Smad3 (1:1,000, # 9,523T), phospho-Smad3 (Ser423/425) (1:1,000, # 9,520), c-Jun (1:1,000, # 9,165), and phospho-c-Jun (Ser63) (1:1,000, # 2,361) were purchased from Cell Signaling Technology (Danvers, MA, United States). Then, the membranes were incubated for 2 h with secondary antibodies (1:2,000, # 7074, Cell Signaling Technology) at 25°C. After the membranes were washed in TBST, chemiluminescent signals were detected using the Bio-Rad Molecular Imager ChemiDoc^TM^ XRS + system (Bio-Rad, United States). GAPDH was used as the loading control.

### Protein 3D Structure Construction and Docking

An online server was used to model the protein sequence, and the template was searched using Homology detection and structure prediction by HMM-HMM comparison^1^ provided by the Max-Planck Institute of Developmental Biology, Germany. The protein sequence was submitted to the HHpred database, the latest PDB database was selected in the database search column, and a template with homology to the target was searched. The single-template modeling script model_singal.py in Modeler 9.23 was used to perform single-template modeling for 3D structure simulation. The energy of the constructed structure was minimized. ZDOCK was used for protein–protein docking, and PyMOL was used to analyse the results before plotting.

### Statistical Analysis

The unpaired, two-tailed Student’s *t*-test was used for comparison between two groups, and one-way analysis of variance (ANOVA) with the Bonferroni *post hoc* test was used for multiple comparisons. For all experiments, *P* < 0.05 was considered significant and indicated by “^∗^”; *P* < 0.01 was indicated by “^∗∗^.”

## Results

### Tartrate Acid Phosphatase-Positive Cell Deficiency Impairs Callus Formation During Bone Healing

**FIGURE 1:**
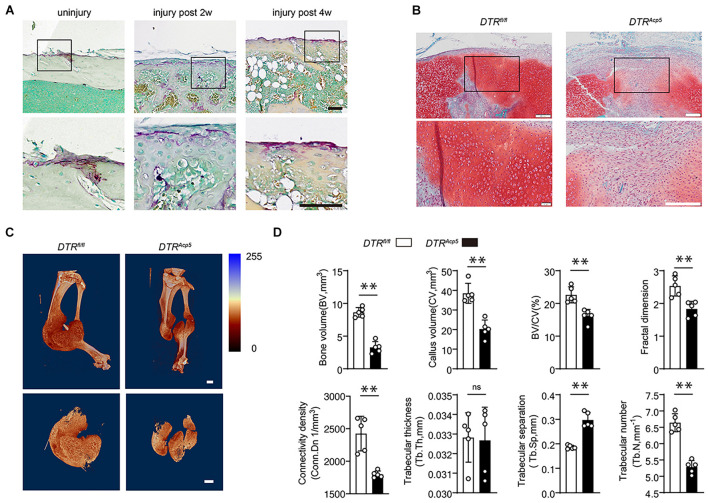
Tartrate-resistant acid phosphatase (TRAP)-positive cell deficiency impairs callus formation during bone healing. **(A)** Localization of TRAP-positive cells in the periosteum and fracture callus during fracture healing in WT mice. Scale bars, 200 μm. **(B)** Representative images of tibia callus Safranin O/Fast green staining on14 days post fractures in WT and *DTR^*Acp*5^* mice. Scale bars, 200 μm. **(C)** Representative 3D micro-computer tomography (μCT) images of tibia bone callus 14 days post fractures. Scale bars, 2 mm. **(D)** Quantitative μCT analysis of tibia fractures in *DTR^*Acp*5^* mice on 14 days post fractures. The μCT parameters includes CV (callus volume), BV (bone volume), BV/CV, FD (Fractal dimension), Tb.Th (trabecular thickness), Tb.Sp (trabecular separation), Tb.N (trabecular number), and Conn.Dn (Connectivity density). Data are expressed as mean ± SD. ^∗∗^*P* < 0.01.

Studies have identified that TRAP-positive cells on the periosteum are mononuclear osteoclast precursors (Xie et al., 2014). However, the precise location of fracture-associated TRAP-positive mononuclear osteoclast precursor remains unknown. To determine the distribution of TRAP-positive cells during fracture healing, we established a closed tibia fracture model in wild-type mice. TRAP staining revealed that TRAP-positive cells increased significantly on the surface of the callus at 2 and 4 weeks after the fracture compared with that at week 0 (Figure 1A).

To further explore the functions of TRAP-positive lineage cells in bone healing, we genetically ablated TRAP-positive cells by crossing Acp5-Cre mice with DTR mice to generate Acp5-Cre; DTR mice (*DTR^*Acp*5^*), which have TRAP-positive cell deficiency after administering diphtheria toxin (DT) injection. The *DTR^*Acp*5^* mice were injected with DT 2 weeks before surgery and analyzed at 2 weeks after the fracture. We induced tibial fractures on 8-week-old *DTR^*Acp*5^* mice and their littermate controls (*DTR*^*fl/fl*^) and found that the mice failed to achieve maximum callus volume at 2 weeks, characterized by delayed cartilage formation leading to fibrosis (Figure 1B). To further investigate mineralized bone callus, we performed μCT analysis. μCT found that the *DTR^*Acp*5^* mice exhibited an impaired bone healing process marked by a decrease in the total fracture callus size and hard bone callus volume at 2 weeks after the fracture compared with the *DTR*^*fl/fl*^ mice (Figure 1C). The VOI volume indicates the total volume of the callus. As shown in Figures 1C,D, the callus volume significantly decreased in the *DTR^*Acp*5^* mice compared with the *DTR*^*fl/fl*^ mice. Other 3D morphometric parameters of μCT including bone volume (BV), callus volume (CV), BV/CV, fractal dimension (FD), connective density (Conn. Dn), trabecular thickness (Tb.Th), trabecular separation (Tb.Sp), and trabecular number (Tb.N), which are sensitive to the connectivity and microstructure of the complex porous callus, were used to evaluate the mineralized callus. As indicated by these morphological parameters, the *DTR^*Acp*5^* mice had significantly reduced bone callus connectivity and microstructure compared with littermate controls (Figure 1D). These results suggest lower quality of the callus in TRAP-positive cell-deficient mice with decreased callus volume and a complex porous callus.

### Connective Tissue Growth Factor From Preosteoclasts Induces Periosteal Stem Cell Renewal and Promotes the Chondrogenic Potential of SSCs *in vitro*

**FIGURE 2:**
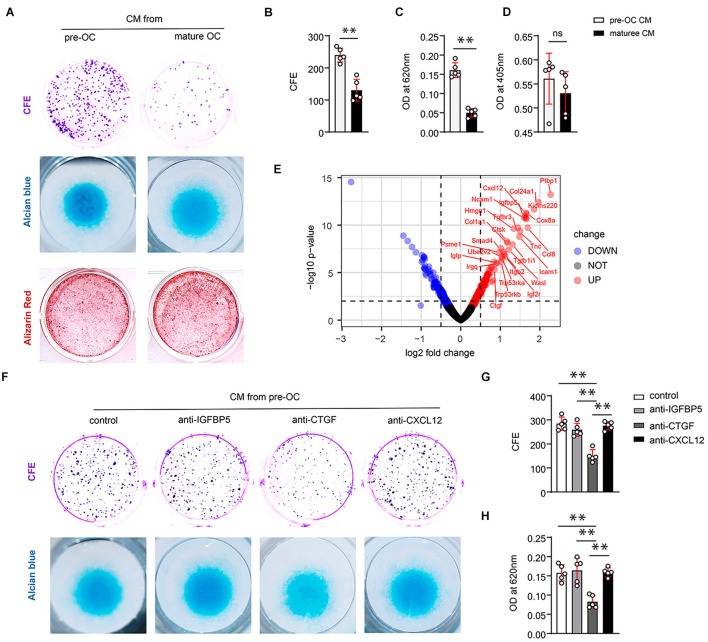
CTGF from preosteoclasts induces periosteal stem cells (PSCs) renewal and promotes the chondrogenic potential of PSCs *in vitro.* **(A)** Representative images of clone formation efficiency (CFE), alcian blue, and alizarin red staining of PSCs. **(B)** Quantitative analysis of the CFE of PSCs. **(C)** Quantitative analysis of alcian blue staining 620 nm absorbance. **(D)** Quantitative analysis of alizarin red staining at 405 nm absorbance. **(E)** Volcano map of differentially expressed proteins in the extracellular matrix after adding TRAP^+^ monocytes. Neutralizing antibodies of IGFBP5, CTGF, and CXCL12 were added to the conditioned medium of preosteoclasts and mature osteoclasts to investigate their effects on PSCs self-renewal and chondrogenesis. **(F)** Representative images of clone formation efficiency (CFE) and Alcian blue. **(G)** Quantitative analysis of the CFE of PSCs. **(H)** Quantitative analysis of Alcian blue staining 620 nm absorbance. Data are shown as mean ± SD. ^∗∗^*P* < 0.01.

PSC activation at an early stage of fracture healing is the basis of callus formation. PSCs exhibit the stem cell properties of clonal multipotency. To explore the possible mechanism of TRAP-positive monocytes (preosteoclasts) in regulating callus formation and PSC activation, we induced differentiation of BMMs into preosteoclasts and mature osteoclasts and collected the different groups of conditioned media. The colony-forming efficiency assay (CFE) was used to detect the cell renewal activity. Compared with the medium derived from mature osteoclasts, the conditioned medium derived from preosteoclasts induced significantly higher PSC clonogenicity (Figure 2A upper panel, Figure 2B), indicating that the factor that promotes PSCs renewal is derived from preosteoclasts. In addition, the conditioned medium derived from preosteoclasts induced significantly more chondrogenesis than that derived from mature osteoclasts (Figure 2A middle panel, Figure 2C). However, the conditioned medium from preosteoclasts rarely affected the osteogenic differentiation activity of PSCs compared with that from mature osteoclasts (Figure 2A lower panel, Figure 2D).

To identify the functional factors, we analyzed protein profile data from a previous study (Dong et al., 2020). In this study, preosteoclasts were introduced to bone tissue engineering as seed cells. iTRAQ-labeled mass spectrometry was performed to analyze the difference between proteins secreted by MSCs alone and MSCs + preosteoclast. Volcano maps were used to show differentially expressed proteins, indicating the secreted protein from preosteoclast (TRAP-positive monocyte) (Figure 2E). Among the differentially expressed proteins, we observed that IGFBP5, CXCL12, and CTGF might be the potential regulators of differentiation and fate of stem cells. To clarify the role of the aforementioned proteins in PSC self-renewal, we tested neutralizing antibodies against IGFBP5, CXCL12, and CTGF in the conditioned media. Only the antibodies against CTGF abolished preosteoclast-induced PSC renewal (Figure 2F upper panel, Figure 2G). Alcian blue staining revealed that only the CTGF neutralizing antibody reduced the chondrogenic ability of the conditioned medium on PSCs (Figure 2F, lower panel, Figure 2H). ELISA revealed that preosteoclasts secreted more CTGF than mature osteoclasts (Supplementary Figures 1A,B). Taken together, our results suggest that preosteoclasts, a TRAP-positive monocyte, secrete CTGF to induce PSC activation after bone fracture and facilitate the bone healing process.

### Connective Tissue Growth Factor From Preosteoclasts Induces Callus Formation During Bone Healing

**FIGURE 3:**
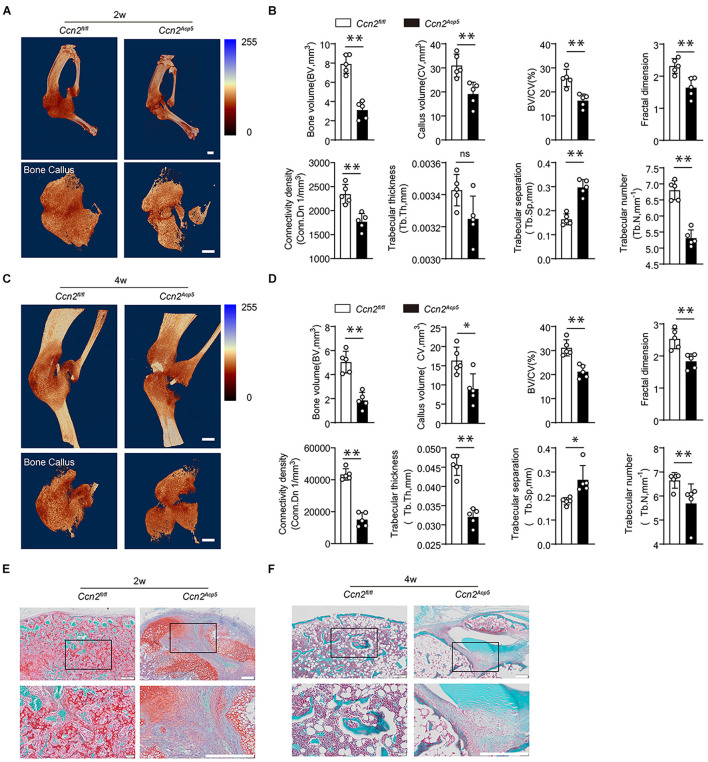
CTGF from preosteoclasts induces callus formation during bone healing. CTGF floxed mice (*Ccn2^*fl*/fl^* mice) were crossed with Acp5-Cre mice to generate TRAP lineage-specific Ccn2 deletion mice (*Acp5-Cre; Ccn2 ^*fl*/fl^*, short as *Ccn2^*Acp*5^*). The tibial fractures were induced on 8-week-old *Ccn2^*Acp*5^* mice and *Ccn2^*fl/fl*^* mice. **(A)** Representative 3D micro-computer tomography (μCT) images of tibia bone callus 14 days post fractures in *Ccn2^*fl*/fl^* and *Ccn2^*Acp*5^* mice. Scale bars, 2 mm. **(B)** Quantitative μCT analysis of **(A)**. **(C)** Representative 3D micro-computer tomography (μCT) images of tibia bone callus 28 days post fractures in *Ccn2^*fl*/fl^* and *Ccn2^*Acp*5^* mice. Scale bars, 2 mm. **(D)** Quantitative μCT analysis of **(C)**. The μCT parameters includes CV (callus volume), BV (bone volume), BV/CV, FD (Fractal dimension), Tb.Th (trabecular thickness), Tb.Sp (trabecular separation), Tb.N (trabecular number), and Conn.Dn (Connectivity density). **(E)** Representative images of tibia callus Safranin O/Fast green staining on 14 days post fractures in *Ccn2^*fl*/fl^* and *Ccn2^*Acp*5^* mice. Scale bars, 200 μm. **(F)** Representative images of tibia callus Safranin O/Fast green staining on 28 days post fractures in *Ccn2^*fl*/fl^* and *Ccn2^*Acp*5^* mice. Scale bars, 200 μm. Data are expressed as mean ± SD. ^∗^*P* < 0.05, ^∗∗^*P* < 0.01.

We next examined the function of CTGF secreted by preosteoclasts in callus formation during bone healing. CTGF floxed mice (*Ccn2^*fl*/fl^* mice) were crossed with Acp5-Cre mice to generate TRAP lineage-specific Ccn2 deletion mice (*Acp5-Cre; Ccn2 ^*fl*/fl^*, short as *Ccn2^*Acp*5^*). We performed immunofluorescence double staining to verify CTGF suppression in TRAP-positive cells in the *Ccn2^*Acp*5^* mice (Supplementary Figure 1C). Tibial fractures were induced in 8-week-old *Ccn2^*Acp*5^* and *Ccn2^*fl/fl*^* mice. The callus size and bone volume were all lower in the *Ccn2^*Acp*5^* mice relative to the *Ccn*^*fl/fl*^ mice (Figure 3). The *Ccn2^*Acp*5^* mice failed to achieve maximum cartilage volume by day 14, leading to non-union at day 28 (Figures 3E,F). To further investigate mineralized callus formation, the μCT analysis was performed. The *Ccn2^*Acp*5^* mice exhibited an impaired bone healing process marked by a decrease in the total fracture callus size and hard callus volume both at week 2 after the fracture compared with the *Ccn2^*fl/fl*^* mice (Figure 3A). Furthermore, the *Ccn2^*Acp*5^* mice had significantly reduced bone callus connectivity and microstructure compared with the *Ccn2^*fl/fl*^* mice, marked by the morphological parameters as previously described in Figures 1D, 3B. The callus usually begins to remodel at 2 weeks after the fracture; however, μCT results showed that bone non-union appeared in the *Ccn2^*Acp*5^* mice 4 weeks after the fracture, which was characterized by a significant decrease in BV and BV/TV (Figures 3C,D). The aforementioned results indicated that CTGF from preosteoclasts induces callus formation by promoting cartilage formation during bone healing, and CTGF depletion in preosteoclasts leads to bone non-union.

### Connective Tissue Growth Factor From Preosteoclasts Is Required to Maintain the Pool of Periosteal Stem Cells and Commit Periosteal Stem Cells to Chondrogenic Lineage

**FIGURE 4:**
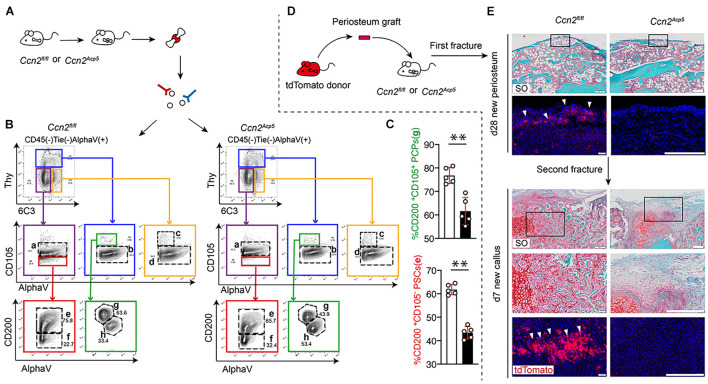
CTGF from preosteoclasts is required to maintain the pool of PSCs and commit PSCs to chondrogenic lineage. After performing fractures on *Ccn2^*Acp*5^* mice and *Ccn2^*fl/fl*^* controls, the callus tissue was dissected and digested for Flow cytometry analysis **(A)**. Representative plots **(B)** and quantification **(C)** of [CD45^–^ Ter-119^–^ Tie2^–^ AlphaV^+^ Thy^–^ 6C3^–^ CD105^–^CD200^+^] PSCs and [CD45^–^ Ter-119^–^ Tie2^–^ AlphaV^+^ Thy^–^ 6C3^–^ CD105^+^CD200^+^] PCPs in the bone callus. **(D)** Schematic diagram of separating the periosteal graft from Tomato donor mice and transplanting it to the fracture site of *Ccn2^*Acp*5^* mice and *Ccn2^*fl/fl*^* controls. **(E)** Representative images of SO staining and DAPI/tdTomato immunofluorescence of fracture tibia after transplantation with tdTomato periosteal graft. In the new periosteum formed 28 days after the fracture, high magnification shows that the scarce periosteum-derived tdTomato^+^ cells are integrated into the new periosteum (white arrow). After the second fracture, a large number of tdTomato^+^ cells derived from periosteum were found in day 7 callus and formed cartilage tissue (white arrow). Scale bars, 200 μm. Data are shown as mean ± SD. ^∗∗^*P* < 0.01.

Studies have reported that [CD45^–^ Ter-119^–^ Tie2^–^ AlphaV^+^ Thy^–^ 6C3^–^ CD105^–^CD200^+^] PSCs exhibit clonal multipotency and self-renewal and sit at the apex of a stem cell differentiation hierarchy in the periosteum (Chan et al., 2015). To investigate the role of CTGF from preosteoclasts in PSC activation after a bone fracture, flow cytometry was used to detect the PSC population (Figure 4A). After establishing a fracture model using 8-week-old *Ccn2^*Acp*5^* mice and their littermate controls, the callus tissue was dissected and digested. The flow cytometry gating strategy of PSCs was based on previously reported literature (Chan et al., 2015). According to the results of flow cytometry, the frequency of CD105^–^ CD200^+^ PSCs in the *Ccn2^*Acp*5^* mice was significantly decreased compared with that in the littermates (Figures 4B,C). As [CD45^–^ Ter-119^–^ Tie2^–^ AlphaV^+^ Thy^–^ 6C3^–^ CD105^+^ CD200^+^] pro-chondrogenic progenitors (PCPs) predominantly gave rise to cartilage with minimal bone and no marrow, considering the importance of chondrogenesis to the callus formation after a bone fracture, we focused on not only the PCPs (subpopulation **e**) but also the PCPs (subpopulation **g**).

To investigate the role of CTGF from preosteoclasts in regulating PSCs and their periosteum niche, we used periosteum transplantation and examined PSC contribution to bone repair after two rounds of fractures (Figure 4D). After the first round of fracture, the *Ccn2^*Acp*5^* mice showed a non-union at day 28, whereas the littermate mice achieved bone healing. After the second round of injury, the littermates formed ossified calluses and new periosteum at day 28, but the *Ccn2^*Acp*5^* mice formed fibrosis callus (Figure 4E). After transplantation of the tdTomato periosteal grafts at the fracture site of wild-type hosts, periosteum-derived tdTomato-positive cells largely contributed to cartilage in the callus, and rare tdTomato-positive cells were localized in the newly formed periosteum by day 28. This indicated that PSCs in the periosteum of the tdTomato donor could be activated after transplantation to continue to participate in the formation of a new periosteum. After the second injury, these rare tdTomato-positive PSCs continued to be reactivated for fracture healing and were detected in the callus cartilage by day 7. When we transplanted the tdTomato periosteal grafts into the *Ccn2^*Acp*5^* hosts, the regenerative ability of PSCs was abolished and could not be detected in the cartilage in the callus, which resulted in failed callus formation and fibrosis. Thus, these data indicate that the non-union phenotype in the *Ccn2^*Acp*5^* mice was due to the impaired PSC renewal activity, and absence of CTGF from preosteoclasts affected the ability of the periosteum to maintain the PSC niche, resulting in obstructed callus formation.

### Connective Tissue Growth Factor From Preosteoclasts Induces Periosteal Stem Cell Renewal and Directs Periosteal Stem Cell Fate via αVβ5

**FIGURE 5:**
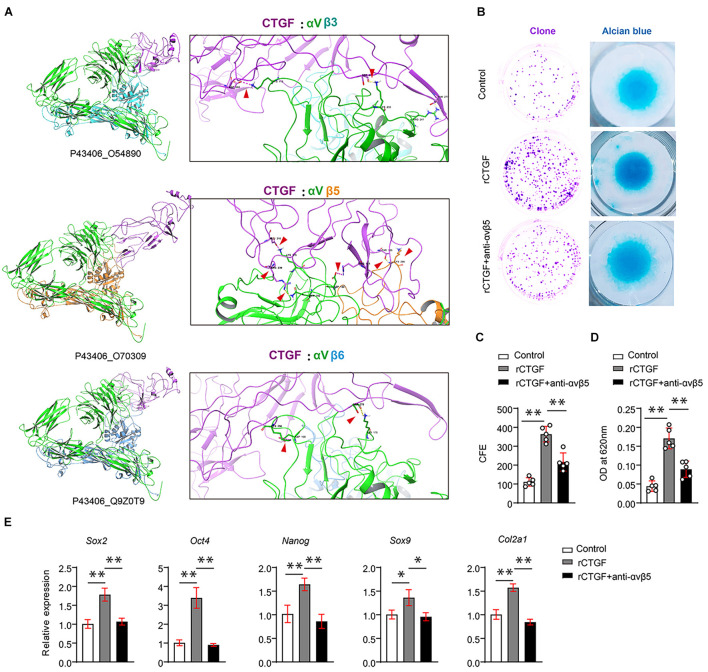
CTGF induces PSCs renewal and chondrogenesis via αVβ5. **(A)** Results of protein docking showed that the ternary complex structure of αVβ5 (P43406_O70309) and CTGF had the largest binding surface, followed by Q9Z0T9, and O54890 was the worst. **(B)** Representative images of clone formation efficiency (CFE) and Alcian blue staining. **(C)** Quantitative analysis of the CFE. **(D)** Quantitative analysis of Alcian blue staining 620 nm absorbance. **(E)** Quantitative PCR analysis of *Sox2*, *Oct4*, *Nanog*, *Sox9*, and *Col2a1* after recombinant CTGF (rCTGF) treatment with or without anti-αVβ5 antibody. Data are shown as mean ± SD. ^∗^*P* < 0.05, ^∗∗^*P* < 0.01.

Although CTGF functions through AlphaV (αV) integrins, αV needs to bind to β subunits, which exist in many forms in the stem cells. αβ integrin interacts with CTGF in the stem cells. To identify which integrin that may have more affinity for CTGF, we used a method that mimics protein molecule docking. First, we performed 3D modeling of protein molecules based on homologous templates according to the protein sequence. The structure of αV integrin (Uniport ID: P43406) was determined by homologous template 3IJE (A chain, similarity: 95%). The structure of CTGF is modeled using templates 5NB8 (similarity: 56%) and 4JPH (similarity: 22%). Then, after modeling the protein structure of CTGF, αVβ3, αVβ5, and αVβ6, protein–protein docking was performed using Zdock. The results revealed that the ternary complex structure of αVβ5 (P43406_O70309) and CTGF had the largest binding surface, followed by those of αVβ6 (Q9Z0T9) and αVβ3 (O54890) (Figure 5A).

To further investigate whether CTGF affects PSC self-renewal and chondrogenesis through αVβ5 integrin, we used αVβ5 integrin neutralizing antibody to block αVβ5 activity. CFE assays demonstrated that the neutralizing antibody partially blocked the effect of CTGF on PSC self-renewal (Figure 5B upper panel, Figure 5C). The results of the alcian blue staining were consistent with those of the CFE assay. Blocking αVβ5 activity significantly reduced the effect of CTGF on PSC chondrogenesis (Figure 5B middle panel, Figure 5D). Next, RT-qPCR was used to detect the expression of pluripotent genes and cartilage marker genes. After CTGF stimulation, αVβ5 blockade significantly decreased the expression of the pluripotent genes *Sox2*, *Oct4*, and *Nanog*. αVβ5 blockade also decreased the expression of the chondrogenic marker genes *Sox9* and *Col2a1* (Figure 5E).

**FIGURE 6:**
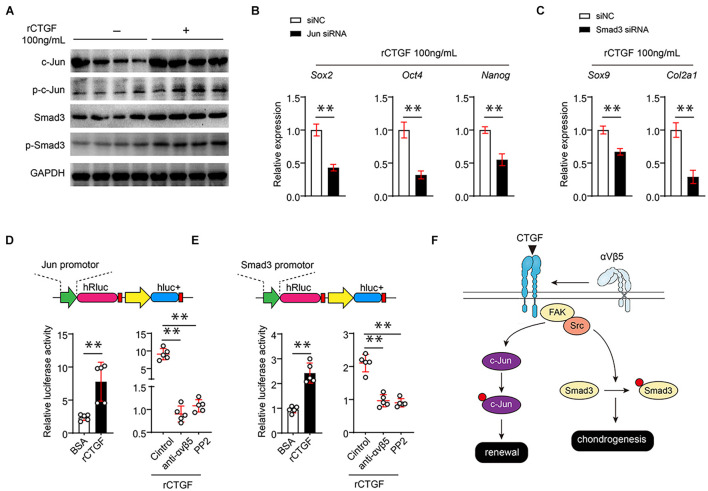
CTGF directly induces the transcriptional activity of c-Jun and Smad3 αVβ5-FAK/Src axis. **(A)** Representative images of western blot of c-Jun, p-c-Jun, Smad3, and p-Smad3 of PSCs treated with or without rCTGF. **(B)** Quantitative PCR analysis of *Sox2*, *Oct4*, and *Nanog* of PSCs treated with rCTGF with or without c-Jun siRNA. **(C)** Quantitative PCR analysis of *Sox9* and *Col2a1* after rCTGF treatment with or without Smad3 siRNA. **(D,E)** The 1,000 bp upstream of the Jun transcription start site (TSE) was cloned in front of the firefly luciferin expression gene (hRluc). rCTGF was added to study its effect on the transcription of Jun and Smad3. At the same time, αVβ5 neutralizing antibody and Src inhibitor PP1 were used to study the effect of αVβ5-FAK/Src axis on the transcription of Jun and Smad3 by rCTGF. The ratio of hRluc to hluc were detected for quantitative statistics. **(F)** Data are shown as mean ± SD. ^∗∗^*P* < 0.01.

αVβ3 directly upregulates the expression of pluripotent genes through c-Jun; therefore, we hypothesized that CTGF affects c-Jun expression through αVβ5. To verify this hypothesis, we first used western blotting to determine whether CTGF directly increases the c-Jun protein level and phosphorylated c-Jun also significantly increases after the introduction of CTGF (Figure 6A). Smad3 is the core controller of chondrocyte differentiation. Western blotting found that CTGF significantly upregulates the protein and phosphorylation levels of Smad3 (Figure 6A). To further investigate whether CTGF functions through c-Jun and Smad3, three different siRNAs targeting c-Jun and Smad3 were introduced into the PSCs. RT-qPCR results showed that the siRNAs targeting *Jun* and *Smad3* effectively reduced the expression of c-Jun and Smad3 (Supplementary Figure 2A). The Western blotting revealed that c-Jun and Smad3 knockdown downregulates pluripotent gene expression and cartilage marker gene expression, respectively (Figures 6B,C). The αβ integrins usually function through the FAK-Src axis. We found that the Src inhibitor PP2 significantly reduced Src phosphorylation caused by rCTGF (Supplementary Figure 2B). To investigate the mechanism of action of CTGF on c-Jun and Smad3 promoter activities, the promoter sequences of c-Jun and Smad3 were cloned into the dual fluorescein reporter gene vector. The αVβ5 blockade and administration of the Src inhibitor PP2 inhibited c-Jun and Smad3 promoter activities, respectively (Figures 6D,E). The mechanism of CTGF on PSCs is shown in Figure 6F.

## Discussion

A new era of bone regeneration-related research has begun with the identification of a series of skeletal stem cells (SSCs) (Chan et al., 2018; Duchamp De Lageneste et al., 2018; Gulati et al., 2018). SSCs in the periosteum that are capable of full-time intramembranous osteogenesis are called PSCs (Debnath et al., 2018). Lineage tracking and renal capsule transplantation experiments have shown that the most important cell source in endochondral ossification in the fracture area is PSCs from the periosteum rather than the BMSCs (Duchamp De Lageneste et al., 2018). PSCs isolated from the periosteum at baseline rarely differentiate into chondrocytes, whereas those isolated from the bone injury site mediate endochondral ossification when transplanted into the kidney capsule model (Chan et al., 2018; Duchamp De Lageneste et al., 2018). This proves that PSCs are activated to mediate endochondral ossification during bone defects (Debnath et al., 2018). This study investigated the mechanism by which PSCs are activated during bone defects. Our results demonstrated that CTGF from TRAP-positive monocytes induces bone callus formation during fracture healing. TRAP-positive monocytes locate at the periosteal and endosteal bone surface through fracture healing. The secreted CTGF directs PSC renewal and commitment to chondrogenic lineage after injury.

The periosteum is a local source of SSCs for bone repair (Debnath et al., 2018; Wang et al., 2019). It is a microvascular connective tissue that covers the outer surface of the cortical bone. The periosteum provides a special microenvironment that promotes bone growth and remodeling (Raggatt et al., 2014). Osteoclasts are abundant on the bone surface. Most osteoclasts in the periosteum are TRAP-positive monocytes and are important for bone remodeling and regeneration. PDGF-BB derived from TRAP-positive precursor osteoclasts on the bone surface can couple angiogenesis in time and space in bone growth and remodeling. TRAP-positive monocyte-conditioned medium induces MSC and EPC migration and enhances angiogenesis. PDGF-BB from periosteal precursor osteoclasts stimulates S1P secretion to promote bone formation, which further couples angiogenesis with bone formation in the periosteal environment (Xie et al., 2014). We achieved similar results and proved that TRAP cells not only play a role in physiological bone remodeling but also promote PSC self-renewal during fracture healing. Our study proves that TRAP cells constitute the niche in the periosteum of PSCs, which are vital for maintaining PSCs in injury and baseline.

CTGF, a CCN family member, performs chemotaxis, induces adhesion, and regulates cell proliferation and migration (Ramazani et al., 2018; Shen et al., 2021). CTGF also regulates bone formation in the cartilage (Takigawa et al., 2003). Moreover, it plays a role in endochondral osteogenesis by regulating almost all types of cells (Takigawa, 2013). CTGF stimulates the proliferation and differentiation of chondrocytes on the growth plate and promotes their hypertrophy to form a calcified matrix. Recombinant CTGF promotes osteoblast proliferation, differentiation, and calcification (Nishida et al., 2000). CTGF promotes the proliferation, adhesion, migration, and lumen formation of vascular endothelial cells (Markiewicz et al., 2011). The main role of CTGF is to promote chondrocyte differentiation. CTGF overexpression in transgenic mice can accelerate endochondral ossification by promoting the proliferation and differentiation of growth plate chondrocytes and promote the increase in the length of their long bones (Tomita et al., 2013). CTGF promotes the differentiation of various chondrocytes, including articular chondrocytes. Another study showed that cartilage-specific CTGF overexpression in transgenic mice can prevent osteoarthritis (Macdonald et al., 2021). Our study proves that CTGF functions by acting on αVβ5 on PSCs. The activated αVβ5 enhances the transcriptional activity of c-Jun and Smad3 through the FAK-Src axis, thereby promoting the expression of pluripotent genes and cartilage marker genes.

The present research has several limitations. The detailed mechanism by which CTGF stimulates PSC activation, such as the cell cycle regulation mechanism, has not been studied. Moreover, high-throughput methods such as gene sequencing and single-cell sequencing should be used to further study the complete molecular mechanism underlying the effect of CTGF on PSCs.

In this study, we demonstrated that TRAP-positive monocytes secrete CTGF to active PSCs during bone regeneration. CTGF from TRAP-positive monocytes promotes endochondral ossification and activates PSCs in mouse bone fracture models. CTGF induces c-Jun expression through αVβ5 integrin, and c-Jun directly activates the transcription of the pluripotent genes *Nanog*, *Sox2*, and *Oct4*. Our research shows that TRAP-positive monocyte-derived CTGF promotes bone healing by activating PSC and directing lineage orientation. Developing technologies targeting PSCs and TRAP-positive monocytes may be an effective strategy to prevent non-union.

## Data Availability Statement

The original contributions presented in the study are included in the article/Supplementary Material, further inquiries can be directed to the corresponding author/s.

## Ethics Statement

The animal study was reviewed and approved by the Laboratory Animal Welfare and Ethics Committee of Third Military Medical University.

## Author Contributions

YB conceived the idea, conducted most of the experiments, and prepared the manuscript. TY collected some specimens and did some of the experiments. TY, SD, and JT helped with processing some of the statistical results. JD and YY provided suggestions for experiments. QD helped in planning the projects and provided suggestions for experiments. SD and JX supervised the project, reviewed, and edited the manuscript. All authors have read and approved the manuscript.

## Conflict of Interest

The authors declare that the research was conducted in the absence of any commercial or financial relationships that could be construed as a potential conflict of interest.

## Publisher’s Note

All claims expressed in this article are solely those of the authors and do not necessarily represent those of their affiliated organizations, or those of the publisher, the editors and the reviewers. Any product that may be evaluated in this article, or claim that may be made by its manufacturer, is not guaranteed or endorsed by the publisher.

## Footnotes

1
http://toolkit.tuebingen.mpg.de/hhpred
