# Supplementary material for: Connective Tissue Growth Factor From Periosteal Tartrate Acid Phosphatase-Positive Monocytes Direct Skeletal Stem Cell Renewal and Fate During Bone Healing
Source: Front Cell Dev Biol. 2021 Sep 14;9:730095. doi: 10.3389/fcell.2021.730095 (PMC8476870; doi:10.3389/fcell.2021.730095)
Supplement: Supplementary file 1 [file Table_1.DOCX]

**Supplemental Materials and Methods**

**ELISA analysis**

The level of CTGF in cell conditioned media was determined by CTGF ELISA kits (# E-EL-M0340, Elabscience, USA).

**Immunofluorescence**

The sections were blocked with 10% BSA and then incubated with anti-CTGF (# sc-365970, Santa Cruz, USA) and anti-TRAP (# M03277-1, Boster Biological Technology, USA) monoclonal antibodies overnight at 4°C. Next, the samples were washed thrice with PBS and probed with secondary antibodies for CTGF with secondary antibody IgGκ BP-CFL 680 (# sc-516180, Santa Cruz) or TRAP (Anti-rabbit IgG Alexa Fluor® 647 conjugated (# 4414, Cell Signaling Technology, USA). The slides were washed thrice with PBS, mounted, and imaged using a Leica SP5 confocal microscope system.

**Real-time quantitative PCR**

PCR amplifications were performed using specific primers for each gene as follows: *Smad3* (F) 5′- CACGCAGAACGTGAACACC- 3′, (R) 5′- GGCAGTAGATAACGTGAGGGA-3′; *Jun* (F) 5′- CCTTCTACGACGATGCCCTC-3′, (R) 5′-GGTTCAAGGTCATGCTCTGTTT-3′.

**Western blots and antibodies**

The primary antibodies for Src (36D10, 1:1000, # 2109) and [phospho-Src (Ser17) (D7F2Q, 1:1000, # 9520)](https://www.cellsignal.com/products/primary-antibodies/phospho-src-ser17-d7f2q-rabbit-mab/12432?site-search-type=Products&N=4294956287&Ntt=src&fromPage=plp) were purchased from Cell Signaling Technology (Danvers, MA, USA). The secondary antibodies (1:2000, # 7074, Cell Signaling Technology) were incubated for 2 h at 25℃.

**Supplementary figure legends**

**Supplementary figure 1**. **TRAP+ monocytes have a higher level of CTGF secretion compared to mature osteoclasts.** (**a**) Schematic diagram of osteoclast induction culture at different stages. (**b**) ELISA analysis of CTGF levels in the conditioned medium from different stages of TRAP-positive monocytes or mature osteoclast. Data are expressed as mean ± SD. **P*<0.05, ***P*<0.01.

**Supplementary figure 2**. **CTGF from preosteoclast was significantly reduced in *Ccn2^Acp2^* mice.** Representative images of TRAP (red) and CTGF (Green) immunofluorescence staining are in *Ccn2^fl/fl^* or *Ccn2^Acp5^* mice. Scale bar, 50μm.

**Supplementary figure 3**. **siRNAs of *Smad3* and *Jun* significantly downregulated the expression of Smad3 and Jun.** Quantitative PCR analysis of *Smad3* and *Jun* after *Smad3* or *Jun* siRNA treatment. Data are shown as mean ± SD. ***P*<0.01.

**Supplementary figure 4**. **Src PP2 significantly blocks Src activation after rCTGF stimulation.** Representative images of western blot of Src and p-Src of PSCs treated with or without rCTGF (100 ng/mL) or PP2 (10 μM).
