# Supplementary figures and images for: Connective Tissue Growth Factor From Periosteal Tartrate Acid Phosphatase-Positive Monocytes Direct Skeletal Stem Cell Renewal and Fate During Bone Healing
Source: Front Cell Dev Biol. 2021 Sep 14;9:730095. doi: 10.3389/fcell.2021.730095 (PMC8476870; doi:10.3389/fcell.2021.730095)

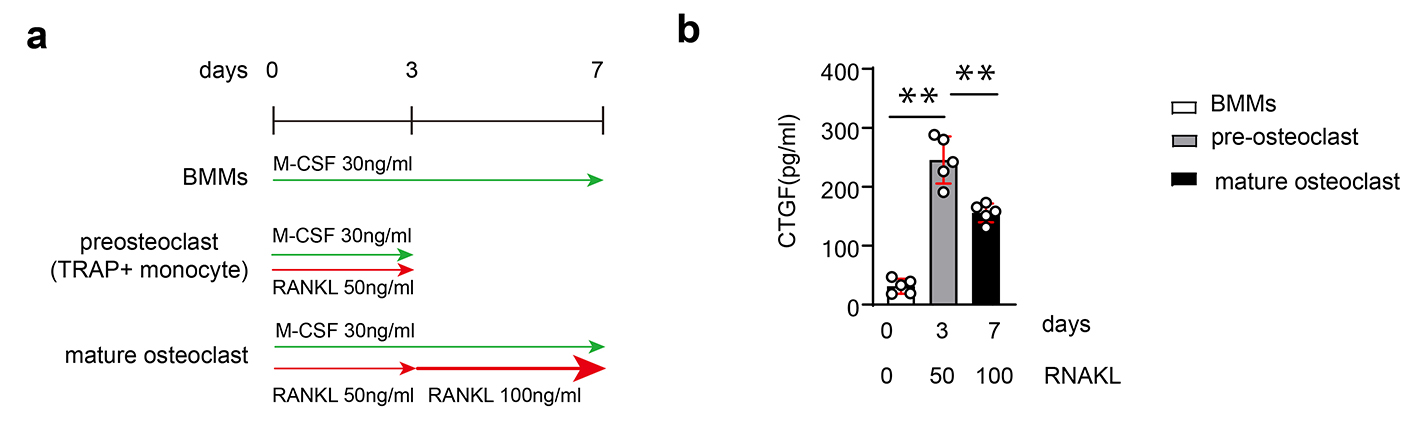

Supplement: Supplementary file 2 [file Image_1.JPEG]

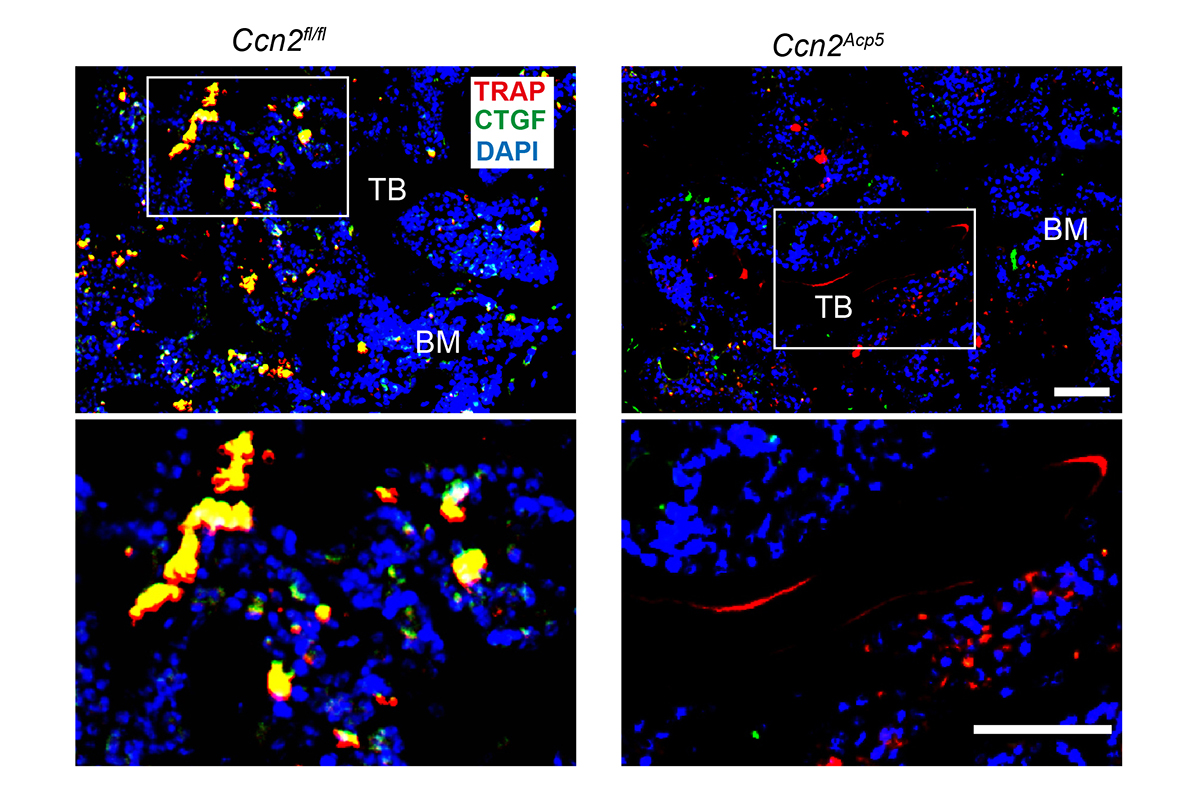

Supplement: Supplementary file 3 [file Image_2.JPEG]

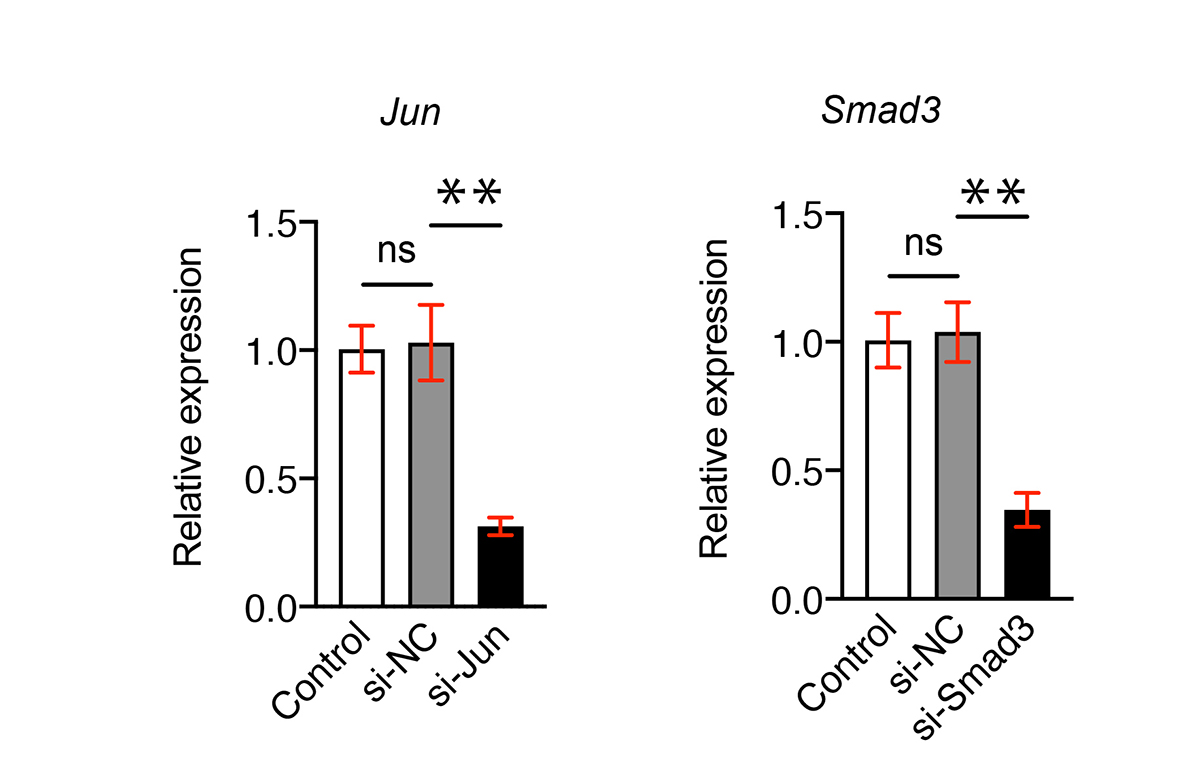

Supplement: Supplementary file 4 [file Image_3.JPEG]

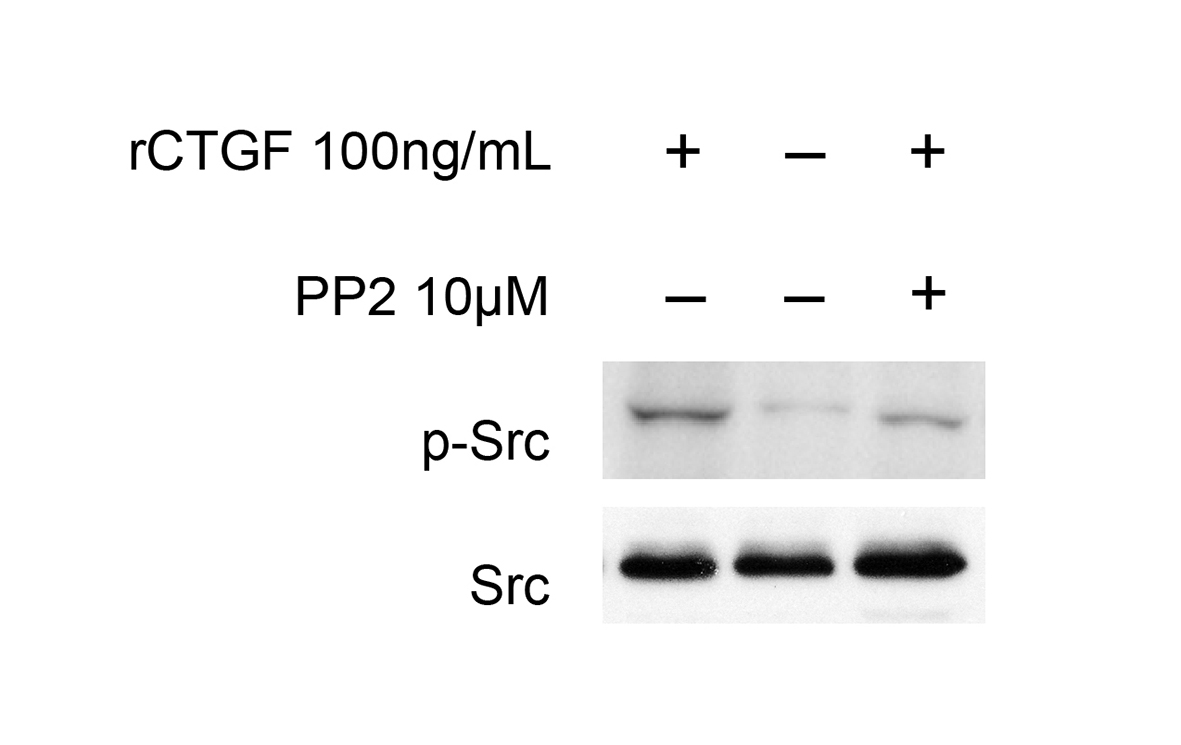

Supplement: Supplementary file 5 [file Image_4.JPEG]
